# Respiratory maneuvers in echocardiography: a review of clinical applications

**DOI:** 10.1186/1476-7120-7-42

**Published:** 2009-08-26

**Authors:** Carmen Ginghina, Carmen C Beladan, Madalina Iancu, Andreea Calin, Bogdan A Popescu

**Affiliations:** 1 "Carol Davila" University of Medicine, Bucharest, Romania; 2 "Prof. Dr. C. C. Iliescu" Institute of Cardiovascular Diseases, Bucharest, Romania

## Abstract

During echocardiographic examination, respiration induces cyclic physiological changes of intracardiac haemodynamics, causing normal variations of the right and left ventricle Doppler inflows and outflows and physiological variation of extracardiac flows. The respiration related hemodynamic variation in intra and extracardiac flows may be utilized in the echocardiography laboratory to aid diagnosis in different pathological states. Nevertheless, physiologic respiratory phases can cause excessive translational motion of cardiac structures, lowering 2D image quality and interfering with optimal Doppler interrogation of flows or tissue motion.

This review focuses on the impact of normal respiratory cycle and provocative respiratory maneuvers in echocardiographic examination, both in physiological and pathological states, emphasizing their applications in specific clinical situations.

## 

Respiration induces cyclic physiological modification of intracardiac haemodynamics. Changes are related to variations in intrathoracic and intraabdominal pressure, systemic and pulmonary venous return, intrapericardial pressure, pericardial constraint and interdependence between the four cardiac chambers. With inspiration intrathoracic and intrapericardial pressures decrease. This results in augmented right ventricular filling and stroke volume and, as the total pericardial space is limited, a compensatory decrease in left ventricular stroke volume occurs in early inspiration. With expiration, intrathoracic and intrapericardial pressures increase, resulting in mild decrease in right ventricular diastolic filling and a subsequent increase in left ventricular filling.

Because of the constraining effect of the pericardium on the combined volume of the four cardiac chambers, respiratory variation in intrapericardial pressure results in reciprocal variation in the filling of both ventricles [1].

## Influence of normal respiration on echocardiographic parameters

### • 2D echocardiography

#### Image quality

With inspiration the anteroposterior diameter of the chest increases, and the lungs inflate and expand particularly anteriorly, partly filling the space between the heart and the thoracic cage. Inspiratory movements do not only decrease the amount of cardiac tissues that lies close to the sternum through anterior pulmonary expansion, but also produce a posterior displacement and rotation of the heart relative to a fixed echo beam [2].

Expiration will give a better parasternal and often apical access to the heart by lungs deflation [see Additional files 1, 2, 3 and 4]. In contrast, in the subxiphoid area inspiration will bring the diaphragm down improving access to the heart [see Additional file 5]. Sometimes half a breath is preferred, as with deep inspiration the abdomen becomes too tense to allow the transducer to be placed under the sternum.

Optimal views are usually obtained with the patient in the steep left lateral decubitus position with the left arm raised to spread the ribs. Rotation of the patient to the left will tend to deflate the left lung avoiding the pulmonary interference [1,3].

#### Measurements

Spontaneous respiration has been shown to cause changes in echocardiographic measurements of left ventricular (LV) dimensions. An inspiratory reduction in LV end-diastolic dimension assessed by M-mode echocardiography was found in normal subjects [4]. As the heart was found to move medially in the short-axis view during inspiration, one possible explanation for this finding could be an inspiratory tangential cut by the M-mode cursor when measured through the center of the left ventricle. Thus the inspiratory dimensions are smaller along the cursor than through the center of the short-axis area [5]. Another possible explanation could be an inspiratory decrease in LV end-diastolic volume and stroke volume as a consequence of preload reduction (decreased diastolic filling) and increased afterload (increased impedance to LV emptying).

Excessive translational motion can be avoided by acquiring images during quiet respiration and in this case several cycles must be recorded. [see Additional files 6, 7] If images are obtained during held end-expiration, care must be taken to avoid a Valsalva maneuver, which can degrade image quality. Image acquisition during quiet or suspended respiration (at end-expiration) is currently recommended for two-dimensional quantitation [3].

### • Doppler echocardiography

#### Physiological respiratory variations of intra and extracardiac flow

**Figure 1:**
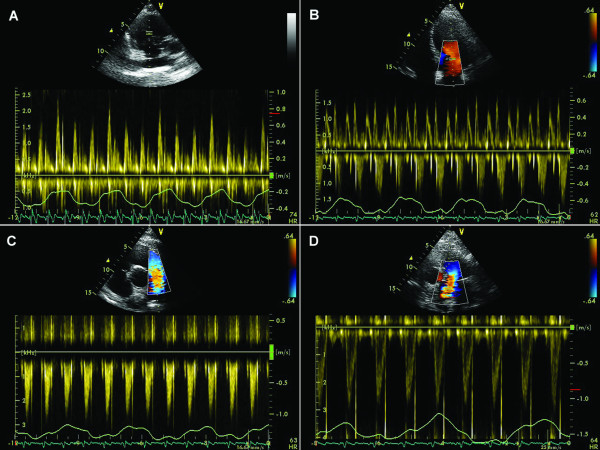
**Physiological variations of the velocity across the tricuspid valve (A), mitral valve (B), pulmonary valve (C) and aortic valve (D) during quiet respiration in a normal heart**.

During inspiration, intrathoracic and intrapericardial pressures decrease, resulting in augmented flow into the right atrium and right ventricle, with decreased flow out of the pulmonary veins into the left atrium and left ventricle. Reciprocal changes occur during expiration, and can be documented by Doppler echocardiographic changes in mitral and tricuspid inflow as well as pulmonary and systemic outflow. Under normal circumstances, peak velocity of mitral inflow varies by 15% or less with respiration and tricuspid inflow by 25% or less. Variation in peak velocity and time velocity integral of aortic and pulmonary flow profiles typically is less than 10%. [1.6](Figure 1)

**Figure 2:**
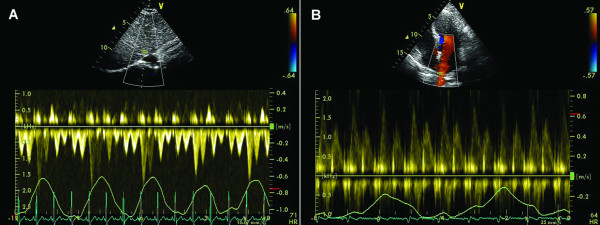
**Physiological variations of the velocity across suprahepatic veins (A) and pulmonary veins (B) during quiet respiration in a normal heart**.

Marked respiratory changes in Doppler velocity curves are observed for hepatic veins. With inspiration there is a significant increase in both normal forward systolic (S) and diastolic (D) flow velocities as well as retrograde A velocity compared to apnea. (Figure 2A).

The normal forward biphasic flow velocity pattern in the superior vena cava (SVC) with systolic flow velocity greater than diastolic flow velocity, shows inspiratory changes similar to those of hepatic vein flow.

Respiratory variations are minimal for pulmonary vein flow (Figure 2B)

**Figure 3:**
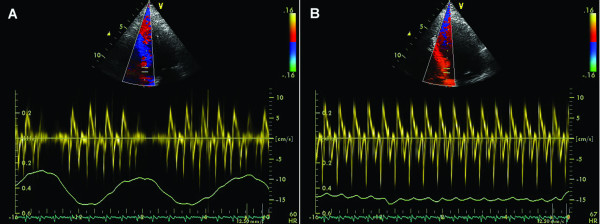
**Septal annular velocities measured by tissue Doppler echocardiography during quiet respiration (A), and during end-expiratory apnea (B)**.

#### Doppler measurements

Diaphragmatic traction associated with respiration changes the position of intracardiac structures in relation to a fixed Doppler sample volume causing errors in velocities measurement.

The Doppler sound beam should be oriented as parallel as possible to the flow, guided both by the 2D image (sometimes assisted by color flow imaging). Small (<20 degrees) deviations in angle produce mild (<10%) errors in velocity measurements. Although these errors may be acceptable for low-velocity flows, when Doppler is used to derive pressure gradients even a small error in velocity measurement can lead to significant underestimation of the gradient because of the quadratic relation between velocity and pressure gradient. The effects of respiration can be minimized by taking measurements during short periods of apnea or by averaging multiple consecutive beats [7,8].

The phase of respiration affects Doppler Tissue Imaging (DTI) recordings in the same manner because the shift in heart position during respiration over a fixed Doppler sample volume leads to inaccurate recording of the annular velocities (Figure 3A).

Whenever possible, the echocardiographer should obtain annular DTI recordings during end-expiratory apnea (Figure 3B). Once the Doppler cursor is aligned optimally and the Doppler tissue preset activated the patient should be asked to breathe in, breathe out, and then hold their breath at the end of expiration. The sample volume will be carefully repositioned directly into the selected portion of the annulus and pulsed wave tissue Doppler activated. Peak DTI waveform velocities should be uniform, consistent, with little or no beat-to-beat variation of the peak velocities [9].

## Respiration and its relationship to cardiac haemodynamic parameters

### • Estimation of right atrial (RA) pressure

**Figure 4:**
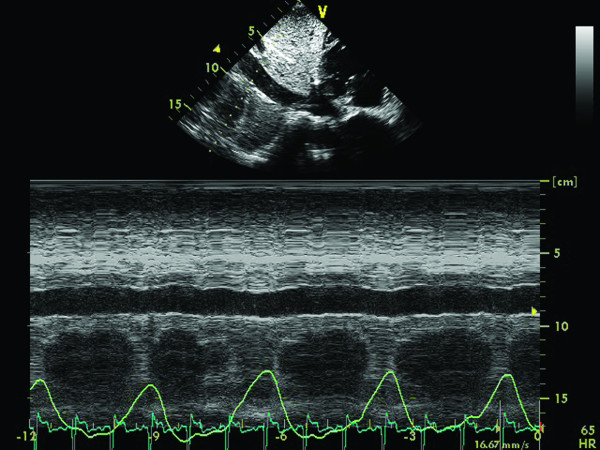
**Patient with dilative cardiomyopathy: M-mode at the inferior vena cava (IVC) level from subcostal view, during respiratory phases**. There is no inspiratory variation of the IVC diameter, indicating increased right atrial pressure.

Evaluation of the inferior vena cava (IVC) from the subcostal view is part of the routine TTE examination. The diameter of the IVC decreases in response to inspiration with minimal size observed at end inspiration when the negative intrathoracic pressure leads to an increase in RV filling from the systemic veins. IVC size is significantly influenced by patient position, being largest in the right lateral position, intermediate in the supine position, and smallest in the left lateral position [10]. The diameter of the IVC, measured with the patient in the left decubitus position at 1.0 to 2.0 cm from the junction with the RA, using the long axis view, and the percent decrease in its diameter during inspiration (collapsibility index) correlate with RA pressure. A brief sniff is often required [see Additional files 8, 9] as normal inspiration may not elicit the inspiratory response [3]:

• The normal IVC diameter is less than 1.7 cm and there is a 50% decrease in the diameter when the RA pressure is normal (0–5 mm Hg).

• A dilated IVC (>1.7 cm) with normal inspiratory collapse (>50%) is suggestive of a mildly elevated RA pressure (6–10 mm Hg).

• When the inspiratory collapse is less than 50%, the RA pressure is usually between 10 and 15 mm Hg.

• Finally, a dilated IVC without any collapse suggests a markedly increased RA pressure of greater than 15 mm Hg. (Figure 4).

A small IVC (usually < 1.2 cm) with spontaneous collapse is often seen in the presence of intravascular volume depletion [11].

In a recent paper, Brennan et al evaluated the IVC diameter in patients undergoing right heart catheterization [12]. The measurements were made from the subcostal view with the patient in supine position. The authors questioned the traditional classification of RAP into narrow 5-mm Hg ranges based on IVC size and collapsibility. They found that the IVC size cutoff with optimum predictive use for RAP above or below 10 mm Hg was 2.0 cm (sensitivity 73% and specificity 85%). They also found that the optimal IVC collapsibility cutoff was 40% (sensitivity 73% and specificity 84%). Defining collapsibility as high (>55%), low (<35%), or normal (35%–50%) and the inferior vena cava as small (<1.7 cm), normal (1.7–2.1 cm), or large (>2.1 cm) the authors suggest a new classification of RAP:

(1) High collapsibility with a small or normal-sized IVC; RAP is very likely low (<5 mm Hg).

(2) High collapsibility with a large IVC or normal collapsibility with a small/normal-sized IVC; RAP is probably between 0 and 10 mm Hg

(3) Normal collapsibility with large IVC; RAP is 10 to 15 mm Hg

(4) Low collapsibility with a large IVC; RAP is clearly high (10–20 mm Hg)

(5) RAP in patients with low collapsibility and a normal-sized or small IVC should be interpreted as indeterminate.

There are several additional conditions to be considered when evaluating the IVC:

• Athletes have been shown to have dilated IVCs with normal collapsibility index

• In mechanically ventilated patients, the relationship between IVC and RAP is controversial; it was shown that a dilated IVC did not always indicate a high RA pressure Nonetheless a small IVC (< 1.2 cm) had a 100% specificity (with a low sensitivity) for a RA pressure of less than 10 mm Hg. It was also suggested that the IVC diameter measured at end expiration and end diastole using M-mode echocardiography correlates better with RA pressure than IVC diameter at end-expiration without ECG synchronization [13].

### •Evaluation of systolic pulmonary artery pressure (PASP)

In patients with chronic obstructive pulmonary disease (COPD) pulmonary hypertension (PH) is sometimes difficult to assess by direct evaluation using the tricuspid regurgitant Doppler velocity because of emphysema or mediastinal deviation. An alternative method of respiration-aided evaluation of PH was proposed by Kunichika et al [14]. Authors found that the expiratory SVC peak systolic forward flow velocity (an index of the RA flow reserve during expiration) is increased in COPD patients with PH, owing to enhancement of the RA flow reserve through the respiratory cycle that compensates for the impaired right ventricular (RV) filling caused by PH. Therefore, respiratory variation in SVC systolic forward flow may be a useful Doppler flow index for the assessment of severity of PH in patients with stable, chronic COPD that cannot been assessed by conventional TTE

### • Assessment of left ventricular diastolic function

Transmitral Doppler flow parameters are widely used in the assessment of LV diastolic function. It is well known that with spontaneous respiration small changes (<15%) in transmitral peak flow velocities occur in healthy subjects. While some authors found no alteration in the ratio of early-to-late peak flow velocity (E/A) [15] other studies reported a significant decrease in E/A ratio with inspiration in normal subjects [16].

The potential effects of spontaneous respiration on mitral inflow Doppler patterns in patients with abnormal LV diastolic function are poorly understood. Tsai et al. [17] observed that during inspiration, the early diastolic peak flow velocity and E/A ratio were reduced in patients with coronary artery disease and abnormal LV relaxation pattern. More recently, Yuan et al. [18] evaluated 51 hypertensive patients presenting with different LV diastolic patterns and observed in 10 of them a characteristic trasmitral Doppler flow phenomenon that displayed E/A ratio > 1 on expiration and < 1 on inspiration. As 8 of 10 patients revealed an early-to-late diastolic tissue velocity ratio <1 authors suggested that this phenomenon could characterize either a new type of diastolic impairment or just pseudo-normal filling unmasked by inspiration. They also suggested that reversed E/A ratio at end-inspiration rather than at end-expiration might be a more sensitive and accurate indicator for abnormal LV diastolic function. Further studies using simultaneous invasive measures are needed to confirm these findings. As the potential effects of respiration on mitral inflow Doppler pattern is still under debate it seems reasonable to take measurements either with simultaneous recording of respiration or during short periods of apnea/by averaging multiple consecutive beats to minimize the effects of respiration.

In diseased ventricles, progressive shortening of the transmitral DT and increasing E/A ratio can be seen with decreasing ventricular compliance and increasing left atrial pressure. Impaired relaxation is frequently masked by elevated filling pressures resulting in a pseudonormal flow pattern (E/A ratio >1). The Valsalva maneuver has been found to effectively unload the heart and unmask an impaired relaxation pattern and high filling pressures in patients with a baseline pseudonormal flow pattern. This maneuver is accomplished first by cessation of breathing at any point in the respiratory cycle when assessment of mitral inflow is optimized. Next, the participant exerts a firm contraction of the abdominal muscles to force air against the closed glottis for approximately 10 seconds thereby increasing the intrathoracic pressure. In order to be properly performed, the Valsalva maneuver should be standardized. This can be done by attaching a mouthpiece (e.g. the tube of a syringe from which the plunger has been detached) to a sphygmomanometer and asking the patient to blow into it in order to raise the column of Hg to 40 mm and keep it stable at this value for 10 seconds [19]. This results in a decrease in both right ventricular and LV filling as a result of an increased thoracic-to-extrathoracic pressure gradient. The mitral inflow pattern is observed for changes during live 2D monitoring of the placement of the Doppler sample. An absolute decrease in the mitral E/A ratio of 0.5 or more can predict an elevated LV filling pressure with a specificity of 100% [20].

Changes in transmitral E/A ratio during the Valsalva maneuver across different stages of diastolic dysfunction were recently described by Reagan et al [21].

**Figure 5:**
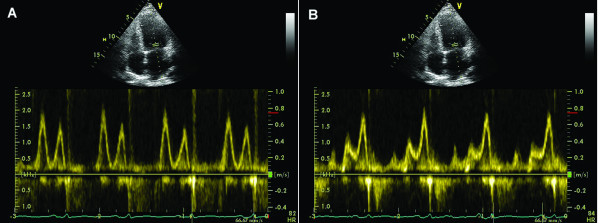
**55 year-old woman, with LV hypertrophy and moderately dilated left atrium**. TTE, apical 4C view, mitral inflow pulse Doppler patterns. A. Mitral inflow pattern appears "normal" at rest. B. During Valsalva maneuver the E/A ratio decreased from 1.3 to 0.6 and peak A velocity increased from 60 to 75 cm/s, unmasking the impaired relaxation pattern.

Because the E/A ratio increases as filling pressures rise it is generally assumed that an E/A ratio <1 (impaired relaxation pattern) indicates lower or even normal filling pressures when compared with patients who have a pseudonormal or restrictive filling pattern. However, Schwammenthal et al demonstrated that patients with an E/A ratio of <1 can nonetheless have severely elevated filling pressures. In these patients LV relaxation is so severely impaired that E/A ratio remains <1 despite increased filling pressures. The standardized Valsalva maneuver can unmask the presence of elevated filling pressures in patients with a baseline impaired relaxation pattern. The A wave will increase during the maneuver in patients with elevated filling pressures in proportion to the level of LV end-diastolic pressure, and independent of the baseline transmitral flow pattern [19].(Figure 5)

## Pericardial diseases and respiration

### • Cardiac tamponade

The increased pericardial pressure in cardiac tamponade produces reciprocal respiration-related changes in right and left ventricular volumes, diastolic filling, and systolic emptying that can be documented by echocardiography. The normal effects of respiration are accentuated such that venous return and right-sided filling occur during inspiration as intrathoracic pressures fall, providing a pressure gradient from the systemic veins to the RA. Because the total intrapericardial volume is fixed by the pressurized effusion, this increased inspiratory RV filling causes the interventricular septum to shift to the left, exaggerating the normal decrease in LV filling volume and, hence, stroke volume (ventricular interdependence). Thus, in tamponade, left heart filling occurs preferentially during expiration when there is less filling of the right heart. The small normal respiratory variation in left ventricular (LV) stroke volume and systolic arterial pressure (<10 mm Hg) is markedly accentuated in cardiac tamponade, resulting in the clinical finding of "paradoxical pulse" [1,22-24].

Echocardiography is particularly useful in demonstrating the exaggerated phasic variation in cardiac volumes and flows caused by tamponade [23]. The diastolic collapse of the free walls of the right atrium and/or right ventricle, due to compression of these relatively low-pressure chambers by the higher-pressure pericardial effusion, is exaggerated during expiration when right heart filling is reduced. The intermittent collapse of these structures is best documented with M-mode echo. [see Additional files 10, 11] Right ventricular collapse may not be seen in patients with PH and RV hypertrophy [25]

Respiratory variation in tricuspid and pulmonary flow is more dramatic than mitral and aortic flow, but there is progressive impairment in all intracardiac flows as the degree of tamponade worsens: with inspiration, the RV early diastolic filling is augmented (>25%), while LV diastolic filling diminishes (>15%). The flow velocity integral in the pulmonary artery increases with inspiration, while the aortic flow velocity integral decreases (>10%) [1].

The hepatic vein flow pattern may also reflect the exaggerated respiratory phase dependency of right ventricular filling. The loss of forward flow in the hepatic veins during the expiration phase of the respiratory cycle with flow out of the hepatic veins confined to the early inspiratory phase can be observed in patients with hemodynamically significant pericardial effusion [1]. Superior vena cava flow shows a reduction in diastolic flow velocities (<0.15 m/sec) initially in expiration, later in inspiration as well (systolic dominant and monophasic) [26]

### • Constrictive pericarditis (CP)

In constrictive pericarditis, the rigid pericardium impedes the transmission of intrathoracic pressures to the cardiac chambers. During inspiration there is a lower driving force from the lungs into the left side of the heart and the LV becomes underfilled. The constricting pericardium also results in an increase interventricular interaction, so that with LV volume decrease, there is a corresponding increase in right ventricular volume [27] [see Additional file 12]

In Doppler echocardiographic studies the dissociation of intrathoracic and intracardiac pressures is manifested by an inspiratory increase in peak tricuspid flow velocity and a simultaneous decrease in mitral flow velocity, with opposite changes occurring in expiration [24].

#### The Doppler findings reflecting abnormal haemodynamics during respiration in constrictive pericarditis are [1,24,28,29]

**Figure 6:**
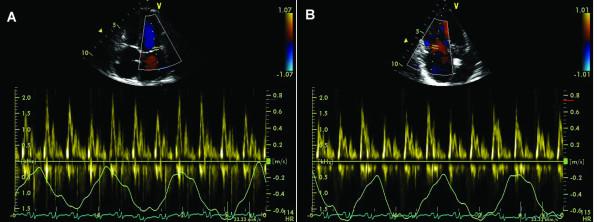
**Constrictive pericarditis**. A (left): respiratory variations of transmitral Doppler inflow: a decrease of peak flow velocity with > 25% (from 2.1 to 1.5 m/s) during inspiration. B (right): respiratory variations of tricuspid inflow (increased flow velocity during inspiration from 1.6 to 1.9 m/s).

**Figure 7:**
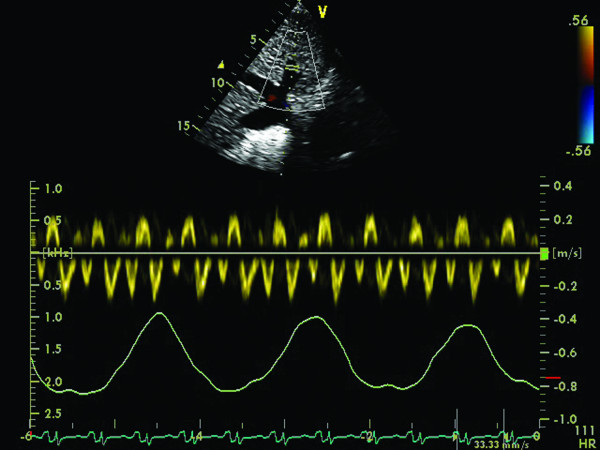
**Constrictive pericarditis**. Respiratory variation of Doppler curve of hepatic vein flow (minimal increase in S and D peak velocities during inspiration, increased A wave during expiration).

• PW Doppler mitral inflow: high E velocity, E/A ratio > 2, short E wave deceleration time (EdT < 160 ms); inspiration: decrease E velocity >25%, prolonged IVRT >25%; expiration: opposite changes (Figure 6A)

• PW Doppler tricuspid inflow: E>A; inspiration: increased tricuspid E velocity >35%, characteristic phenomenon increased TR velocity (Figure 6B)

• PW Doppler recordings of hepatic vein flow: inspiration-minimally increased S and D; expiration: decreased diastolic flow/exaggerated atrial reversal waves (Figure 7)

• PW Doppler recordings of pulmonary vein flow: S/D ratio = 1, inspiration: decreased PV S and D waves, expiration: opposite changes

• SVC Doppler usually shows a diastolic dominant pattern, minimal respiratory variation as right atrial pressure is constantly elevated throughout the respiratory cycle by the thickened, constraining pericardium

• Inspiration: aortic velocity decreases (-14 ± 5%), pulmonary artery velocity increases (16 ± 4%)

• Dilated IVC with reduced inspiratory change in diameter

• Respiratory changes in the proximal aortic waveform in constrictive pericarditis, similar to those described in the LV outflow tract and transmitral pattern, are reported by some authors [30]: an inspiratory decrease and an expiratory increase in forward systolic flow.

There are some clinical and hemodynamic situations that cause difficulties in revealing the significant respiratory Doppler findings for constrictive pericarditis as follows:

#### False negative respiratory variations

• In patients with ***notably elevated left atrial pressure***, respiratory variation of the Doppler inflows may not be present unless preload is reduced by head-up tilt or diuretics [31]

• ***Not all patients ***with surgically proven constriction actually have ***increased respiratory variation of Doppler inflows ***[32].

• In patients with ***atrial fibrillation ***some authors [33] reported that evaluation of respiratory variation in pulmonary vein flow could be more helpful in diagnosing CP than mitral valve inflow, because there is greater respiratory variation in the D wave velocity variables than in the E velocity variables.

#### False positive respiratory variations

• The same pattern of constriction, with respiratory variations, can persist for variables time periods ***after pericardiectomy***

• Patients with ***chronic obstructive pulmonary disease (COPD) ***[34] without constriction may have increased respiratory variation in mitral and tricuspid inflow velocities due to increased change in intrathoracic pressure with respiration. In contrast to constriction, variation is not typically maximal on the first inspiratory beat. In addition, mitral inflow is less restrictive with lower E/A ratios and longer deceleration times than in patients with constriction. Analysis of the SVC Doppler signal may be useful in this setting. SVC Doppler shows pronounced increase in forward flow with inspiration in patients with COPD which is not usually seen in constriction. In addition, patients with COPD usually have systolic dominant SVC Doppler flow rather than diastolic dominance seen in constriction.

**Respiratory maneuvers are very useful in echocardiographical differential diagnosis constrictive pericarditis (CP) – RCM (restrictive cardiomyopathy) as follows **(Table 1) [28]

**Table 1:** Comparison of respiratory changes in mitral, pulmonary vein, tricuspid, and hepatic vein flow in patients with constrictive pericarditis vs restrictive cardiomyopathy

	RCM	CP
Mitral inflow	No respiration variation of mitral inflow E wave velocity, IVRT E/A ratio >2, short DT, diastolic regurgitation	Inspiration: decreased inflow E wave velocity, prolonged IVRT Expiration: opposite changes, short DT, diastolic regurgitation

Pulmonary vein	Blunted S/D ratio (0.5), prominent and prolonged AR No respiration variation, D wave	S/D ratio = 1, Inspiration: decreased pulmonary vein S and D waves Expiration: opposite changes

Tricuspid inflow	Mild respiratory variation of tricuspid inflow E wave velocity, E/A ratio >2, TR peak velocity, no significant respiration change Short DT with inspiration, diastolic regurgitation	Inspiration: increased tricuspid inflow E wave velocity, increased TR peak velocity Expiration: opposite changes Short DT, diastolic regurgitation

Hepatic veins	Blunted S/D ratio, increased inspiratory reversals	Inspiration: minimally increased hepatic veins S and D Expiration: decreased diastolic flow/increased reversals

E, early rapid filling wave; A, filling wave due to atrial contraction; IVRT, isovolumic relaxation time; DT, deceleration time; S, systolic flow; D, diastolic flow; TR, tricuspid regurgitation

## Mixed pathology: restriction and constriction [35]

• High respiratory variation in tricuspid and mitral flow velocities, one of the major criteria to confirm constrictive physiology are decreased in patients with mixed pathology (CP and RCM) because ventricular filling is limited mainly by a noncompliant restrictive myocardium rather than a constrictive pericardium. In addition, these patients usually have markedly increased left atrial and pulmonary venous pressure. Thus, the normal inspiratory intrathoracic pressure decline may cause minimal change in pulmonary venous and left atrial pressure.

## Differential diagnosis for the respiratory variations of diastolic pattern

• Similar respiratory variation in Doppler mitral E wave velocity are caused, as shown, by ***cardiac tamponade***, ***constrictive pericarditis***, ***chronic obstructive pulmonary disease***, but also by ***acute right ventricular dilatation ***due to ***right ventricular infarction or pulmonary embolism***. Most of these conditions can be distinguished by clinical and morphological echocardiographic features (i.e., presence of a pericardial effusion or markedly dilated right ventricle.

### • Valsalva maneuver in the diagnosis of atrial shunt and its functional significance

A patent foramen ovale (PFO) can be reliably detected with contrast echocardiography, using agitated saline; the transthoracic and transesophageal echocardiography evaluation for detecting a patent foramen ovale is performed during normal respiration and during Valsalva maneuver During Valsalva, atrial shunting from right to left will begin during the release phase (phase III) [36]. The Valsalva maneuver may enhance atrial level shunting because of increase in right heart pressure, making the shunt detectable by either transthoracic echocardiography (TTE) or, with a better sensitivity, by transesophageal echocardiography (TEE). From a clinical point of view, it appears that a resting PFO may have a higher risk for clinical events than a PFO with shunt only during a provocative maneuver.

The right-to-left shunt of a large atrial septal defect may be nearly continuous, whereas for smaller atrial septal defects the appearance of contrast in the left atrium may be phasic, coordinated with the respiratory cycle; during inspiration, right heart filling increases, thus increasing the flow of contrast into the right atrium.

### Dynamic obstruction of hypertrophic cardiomyopathy during Valsalva

**Figure 8:**
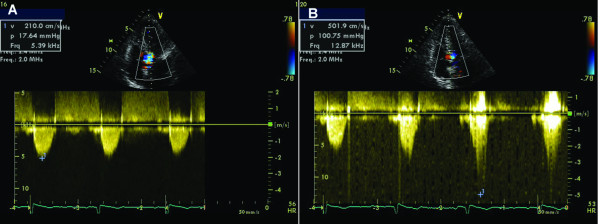
**67 year-old female, with severe hypertrophic cardiomyopathy (resting LV outflow tract gradient of 170 mm Hg at admission)**. A. After beta-blocker treatment, resting CW trace revealed a LV outflow tract peak velocity of 2.1 m/s, with a corresponding gradient of 18 mmHg; B. During Valsalva maneuver, peak velocity and gradient increased to 5 m/s, and 101 mm Hg, respectively.

In patients with obstructive hypertrophic cardiomyopathy the obstruction of the LV outflow tract is dynamic and may be mild or nonexistent at rest. Echocardiography during Valsalva maneuver can unmask latent gradients in patients without resting obstruction and reveal the basis for drug treatment choices. During the strain phase of Valsalva maneuver, due to the decrease in preload, end-diastolic left ventricular volume and afterload, SAM occurs earlier in systole, mitral-septal contact lasts longer and left ventricular outflow tract gradient increases (Figure 8).

#### • Valsalva for assessing mitral regurgitation in mitral valve prolapse

Due to left ventricular volume decrease during Valsalva maneuver in patients with mitral valve prolapse mitral regurgitation starts earlier in systole and severity of mitral regurgitation increases.

#### • Műler maneuver

The Mûller maneuver, the opposite of Valsalva, less often used in echo examinations, is performed by forcibly inspiring while the nose is held closed and the mouth is sealed for about 10 seconds. Because the maneuver exaggerates inspiratory effort, right-sided filling is augmented and can be use for the augmentation of tricuspid regurgitation.

## Limitations

• In the absence of normal sinus rhythm, respiratory variation of Doppler flow cannot be assessed. An irregular heart rhythm does not allow a meaningful interpretation of the influence of respiration on transvalvular Doppler flow.

• The Valsalva maneuver should only be performed in a standardized manner. Lack of standardization of the Valsalva maneuver can limit its feasibility and, more importantly, can lead to wrong conclusions.

• The use of Valsalva maneuver is limited in certain conditions: lack of patient' cooperation; patient's inability to adequately increase intrathoracic pressure because of medical illness, recent operation, sedation (eg during TEE).

• The decrease in image quality sometimes encountered during Valsalva maneuver or with normal respiration may interfere with the interpretation of the findings. However, appropriate training and regular use of respiratory maneuvers help in this regard.

## Conclusion

Using normal respiratory phases and provocative respiratory maneuvers (e.g. Valsalva) can be very useful in characterizing normal cardiac function parameters as well as various cardiac disorders (Table 2). The extra time required to perform these maneuvers is worthwhile and well compensated by the significant increase in examination quality and diagnostic accuracy.

**Table 2:** When, why and how to use normal respiration or Valsalva maneuver during an echo study

**WHEN**	**WHY**	**HOW**
**Poor 2D image quality**	To optimize the quality of the echo view	**Expiration:** better parasternal and often apical access to the heart
		**Inspiration:** brings the diaphragm down improving access to the heart

**M-mode measurements of the LV or 2D-quantitation**	To avoid measurement errors due to excessive translational motion of the heart	**Quiet respiration/held end-expiration**

**Doppler measurements (flow/tissue velocities)**	To avoid measurement errors due to excessive translational motion of the heart	**End-expiratory apnea**

**Estimation of right atrial (RA) pressure**	To elicit the inspiratory response of the inferior vena cava in order to assess the collapsibility index	**Inspiration/brief sniff**

**Evaluation of systolic pulmonary artery pressure in patients with stable, chronic COPD that cannot been assessed by conventional TTE**	To assess the respiratory variation in superior vena cava (SVC) systolic forward flow	**Normal respiration**

**Assessment of left ventricular diastolic function**	To unmask elevated LV filling pressure in patients with normal or reduced LV systolic function and	**Standardized Valsalva maneuver**
	pseudo-normal filling pattern at baseline	decrease in the mitral E/A ratio of 0.5 or more during Valsalva
	impaired relaxation pattern at baseline	increase in peak A wave velocity during Valsalva

**Cardiac tamponade**	To assess respiratory variation in cardiac volumes and flow (see text)	**Normal respiration**

**Constrictive pericarditis**	To assess respiratory variation in mitral, tricuspid, pulmonary and hepatic vein flow (see table 1)	**Normal respiration**

**Restrictive cardiomyopathy**	To assess respiratory variation in mitral, tricuspid, pulmonary and hepatic vein flow (see table 1)	**Normal respiration**

**Detection of Patent Foramen Ovale by TTE or TEE**	To assess the appearence of contrast in the LA shortly after injection of saline contrast into an upper extremity vein, with good opacification of the RA	**Normal respiration** within 3 cardiac cycles after injection of saline contrast
		**Valsalva maneuver** during the release phase

**Hypertrophic cardiomyopathy with mild or absent resting obstruction**	To unmask latent gradients/to increase LVOT gradient	**Valsalva maneuver** during the strain phase

LV, left ventricle; RA, right atrium; SVC, superior vena cava; COPD, chronic obstructive pulmonary disease; TTE, transthoracic echocardiography; E, peak early diastolic velocity oF mitral inflow; A, peak late diastolic velocity of mitral inflow; TEE, transesophageal echocardiography; LA, left atrium; LVOT, left ventricular outflow tract.

## Competing interests declaration

The authors declare that they have no competing interests.

## Authors' contributions

CG conceived of the review, participated in its coordination and revised the final draft of the manuscript. CCB revised the literature, prepared the final draft of the manuscript and provided echocardiographic illustrations. MI revised the literature and prepared the first draft of the manuscript. AC revised the manuscript critically for important intellectual content. BAP participated in the coordination of this review and revised the manuscript critically for important intellectual content. All authors read and approved the final manuscript.

## Supplementary Material

Additional file 1 **Changes in the quality of the echocardiographic image with inspiration**. Transthoracic parasternal long axis view of the left ventricle recorded with normal respiration revealing a decrease in the quality of the echocardiographic image with inspiration. Click here for file

Additional file 2 **Improvement in the quality of the echocardiographic image recorded during held end expiration**. Transthoracic parasternal long axis view of the left ventricle recorded during held end expiration showing a significant improvement in the quality of the echocardiographic image. Click here for file

Additional file 3 **Influence of pulmonary interference on echocardiographic visualization of cardiac structures during normal respiration**. Transthoracic apical 4 chamber view recorded during normal respiration. During inspiration pulmonary interference does not allow the visualization of cardiac structures. Click here for file

Additional file 4 **Influence of expiration on echocardiographic visualization of cardiac structures**. Transthoracic apical 4 chamber view. Expiration allows apical access to the heart by lung deflation. Click here for file

Additional file 5 **Influence of inspiration on echocardiographic image in subcostal view**. Subcostal 4 chamber view. Inspiration brings the diaphragm down, improving imaging of the heart. Click here for file

Additional file 6 **Excessive translational motion of the heart with normal respiration**. Transthoracic short axis view recorded during normal respiration. The heart moves medially with inspiration. Click here for file

Additional file 7 **Image acquisition during suspended respiration (held end-expiration) in a patient with excessive translational motion of the heart with normal respiration**. Transthoracic short axis view. Excessive translational motion can be avoided by acquiring images during held end-expiration. Click here for file

Additional file 8 **Lack of variation of the inferior vena cava diameter during deep inspiration in a patient with dilated cardiomyopathy and severe pulmonary hypertension**. The subcostal view adjusted to demonstrate the long axis of the inferior vena cava (IVC) in a patient with dilated cardiomyopathy and severe pulmonary hypertension. There is no inspiratory variation of the IVC diameter during deep inspiration. Click here for file

Additional file 9 **Lack of variation of the inferior vena cava diameter during brief sniff in a patient with dilated cardiomyopathy and severe pulmonary hypertension**. Evaluation of the inferior vena cava (IVC) from the subcostal view during brief sniff in a patient with dilated cardiomyopathy and severe pulmonary hypertension demonstrates no variation of the IVC diameter. Click here for file

Additional file 10 **Phasic variation in cardiac volumes visualized in transthoracic basal short axis view caused by cardiac tamponade**. Transthoracic basal short axis view in a patient with cardiac tamponade demonstrates diastolic collapse of the free walls of the right atrium and right ventricle. Click here for file

Additional file 11 **Phasic variation in cardiac volumes caused by cardiac tamponade, visualized in transthoracic apical 4 chamber view**. The intermittent collapse of the free walls of the right atrium and right ventricle visualized in the transthoracic apical 4 chamber view in a patient with cardiac tamponade. Click here for file

Additional file 12 **Increased interventricular interaction in a patient with constrictive pericarditis**. Transthoracic apical 4 chamber view in a patient with constrictive pericarditis reveals the increased interventricular interaction (with left ventricular volume decrease there is a corresponding increase in right ventricular volume). Click here for file
